# Power generation of the midfoot in children wearing sports shoes

**DOI:** 10.1186/1757-1146-6-S1-O35

**Published:** 2013-05-31

**Authors:** Caleb Wegener, Andrew Greene, Joshua Burns, Benedicte Vanwanseele, Adrienne E Hunt, Richard M Smith

**Affiliations:** 1 Discipline of Exercise and Sport Science, Faculty of Health Sciences, The University of Sydney, NSW, 1825, Australia; 2 Faculty of Health, Social Care & Education, Anglia Ruskin University, Chelmsford, CM1 1SQ, England, UK; 3 The University of Sydney and The Children’s Hospital at Westmead, Sydney, Australia; 4 Department of Kinesiology, KULeuven, Leuven, Belgium/Chair Health Innovation and Technology, Fontys University of Applied Sciences, Eindhoven, the Netherlands

## Background

During propulsion of walking the midfoot generates 35 to 48% of the peak power from the foot and ankle. This study aimed to investigate the effect of children’s sports shoes on midfoot kinetics during propulsion of walking and running.

## Methods

Twenty children performed five walking and running trials at a self-selected velocity while barefoot and wearing a common sports shoe. Footwear testing order was randomised. A 14 camera motion analysis system was used to calculate retro-reflective marker trajectories at 200Hz. Markers were attached to the leg and to the foot through holes in the shoe to measure three-dimensional motion of the midfoot and ankle. Ground reaction force data were recorded at 1,000Hz. Data were normalised to the stance phase and analysed from 60% to 100%.

## Results

Peak midfoot power generation during walking reduced from 1.67W/kg (SD 0.59) barefoot to 0.50W/kg (SD 0.26) in the sports shoe (*P*<0.0005). Peak ankle power generation during walking was increased from 1.49W/kg (SD 0.42) barefoot to 1.89W/kg (SD 0.44) in the sports shoe (*P*<0.0005). Peak midfoot power generation during running was significantly reduced from 3.92W/kg (SD 1.33) barefoot to 1.56W/kg (SD 0.76) in the sports shoe (*P*<0.0005). Peak ankle power generation during running increased from 4.77W/kg (SD 1.02) barefoot to 6.03W/kg (SD 1.14) in the sports shoe (*P*<0.0005).

## Conclusion

Children compensate for a reduction in midfoot power generation in sports shoes by increasing ankle power generation with potential implications for overuse of the Achilles tendon and triceps surae muscle complex.

